# Knockdown of sarcolipin (SLN) impairs substrate utilization in human skeletal muscle cells

**DOI:** 10.1007/s11033-022-07387-0

**Published:** 2022-04-02

**Authors:** Abel M. Mengeste, Parmeshwar Katare, Andrea Dalmao Fernandez, Jenny Lund, Hege G. Bakke, David Baker, Stefano Bartesaghi, Xiao-Rong Peng, Arild C. Rustan, G. Hege Thoresen, Eili Tranheim Kase

**Affiliations:** 1 Section for Pharmacology and Pharmaceutical Biosciences, Department of Pharmacy, University of Oslo, P.O. Box 1068 Blindern, 0316 Oslo, Norway; 2 Bioscience Metabolism, Research and Early Development Cardiovascular, Renal and Metabolism, BioPharmaceuticals R&D, AstraZeneca, Gothenburg, Sweden; 3 Department of Pharmacology, Institute of Clinical Medicine, University of Oslo, Oslo, Norway

**Keywords:** Obesity, Skeletal muscle, SERCA, Sarcolipin, Glucose metabolism, Lipid metabolism

## Abstract

**Background:**

Recent studies have highlighted that uncoupling of sarco-/endoplasmic reticulum Ca^2+^-ATPase (SERCA) by sarcolipin (SLN) increases ATP consumption and contributes to heat liberation. Exploiting this thermogenic mechanism in skeletal muscle may provide an attractive strategy to counteract obesity and associated metabolic disorders. In the present study, we have investigated the role of SLN on substrate metabolism in human skeletal muscle cells.

**Methods and results:**

After generation of skeletal muscle cells with stable SLN knockdown (SLN-KD), cell viability, glucose and oleic acid (OA) metabolism, mitochondrial function, as well as gene expressions were determined. Depletion of SLN did not influence cell viability. However, glucose and OA oxidation were diminished in SLN-KD cells compared to control myotubes. Basal respiration measured by respirometry was also observed to be reduced in cells with SLN-KD. The metabolic perturbation in SLN-KD cells was reflected by reduced gene expression levels of peroxisome proliferator-activated receptor γ coactivator 1α (*PGC1α*) and forkhead box O1 (*FOXO1*). Furthermore, accumulation of OA was increased in cells with SLN-KD compared to control cells. These effects were accompanied by increased lipid formation and incorporation of OA into complex lipids. Additionally, formation of complex lipids and free fatty acid from de novo lipogenesis with acetate as substrate was enhanced in SLN-KD cells. Detection of lipid droplets using Oil red O staining also showed increased lipid accumulation in SLN-KD cells.

**Conclusions:**

Overall, our study sheds light on the importance of SLN in maintaining metabolic homeostasis in human skeletal muscle. Findings from the current study suggest that therapeutic strategies involving SLN-mediated futile cycling of SERCA might have significant implications in the treatment of obesity and associated metabolic disorders.

**Graphical abstract:**

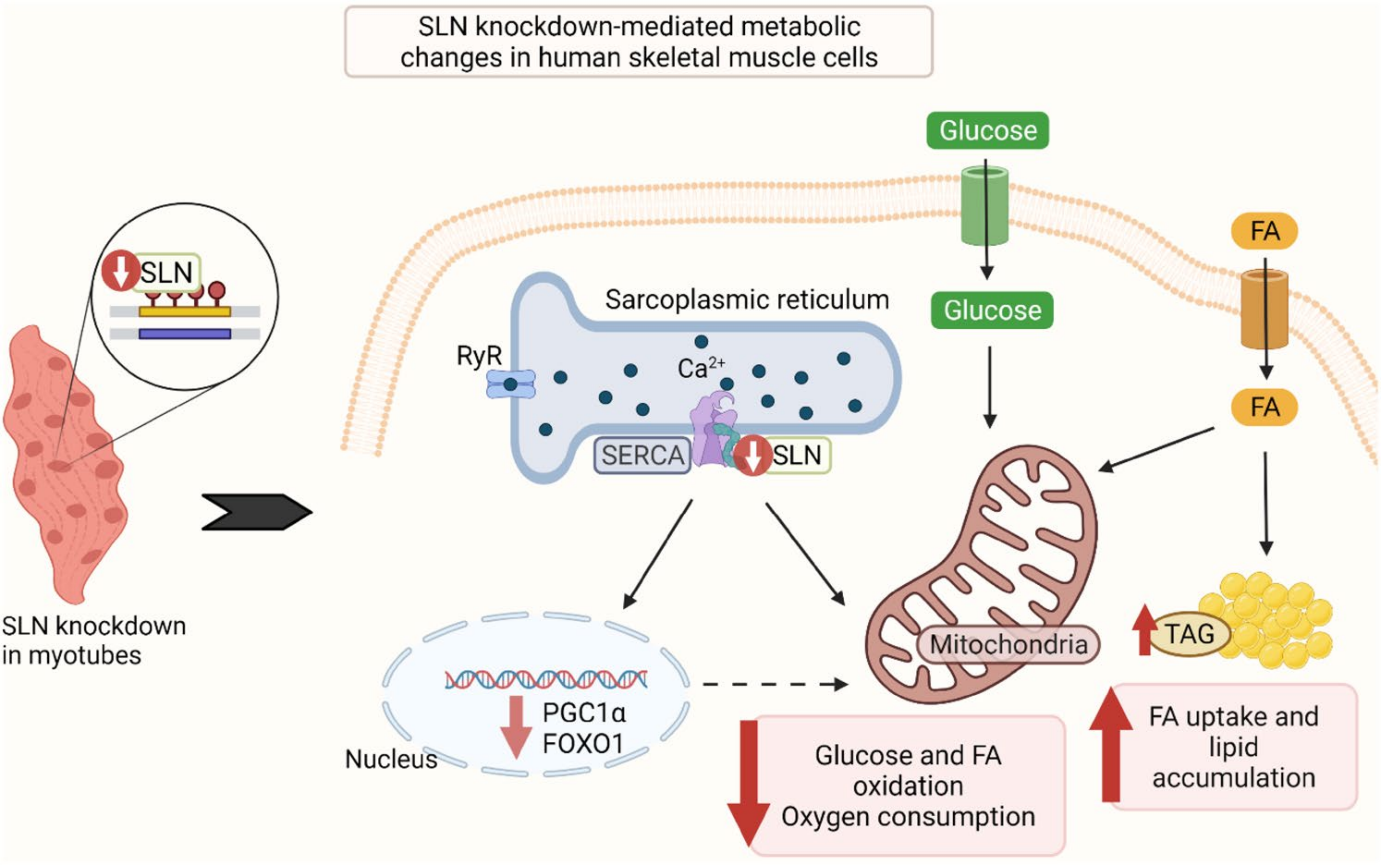

## Introduction

The rising prevalence of obesity in both developed and undeveloped countries remains one of the greatest unsolved public health problems [1]. Obesity is generally characterized by an excess accumulation of body-fat and is often associated with disruption in energy balance, where energy intake exceeds total body energy expenditure [2, 3]. Thus, any treatment for obesity must impact energy intake, energy output, or have an effect on both. However, current available treatment options [4–6] focuses mainly on reducing energy intake. Therapeutic strategies aiming to increase energy expenditure may therefore offer an alternative approach which complement the current obesity management. For this reason, targeting thermogenic mechanisms in skeletal muscle has received more attention due to its potential to increase cellular energy expenditure [3, 7, 8].

Skeletal muscle is an important site of heat production in the body and can generate heat through shivering and non-shivering mechanism, both of which are highly energy demanding processes [9–11]. Although the mechanism responsible for heat production due to repetitive muscle contraction (shivering) is known, energy utilization independent of muscle contraction is still under investigation. Different studies have identified sarco-/endoplasmic reticulum Ca^2+^-ATPase (SERCA) as one of the main contributors for skeletal muscle non-shivering thermogenesis (NST) [9, 12–14]. SERCA is an ATP-driven pump that actively transport Ca^2+^ ions from the cell lumen into the sarcoplasmic reticulum (SR) and plays an important role in maintaining cytosolic Ca^2+^ ion concentration [15]. Maintenance of Ca^2+^ ion gradient in skeletal muscle by the SERCA pump utilize significant amounts of ATP even in resting skeletal muscles, which contribute to more than 40% of the resting metabolic rate [16].

Several lines of evidence suggests that the activity of SERCA is regulated by sarcolipin (SLN) [17–21]. The binding of SLN to SERCA disengages ATPase activity from Ca^2+^ transport, promoting futile cycling of SERCA which leads to increased ATP hydrolysis and heat generation [18, 20, 21]. In addition to muscle thermogenesis, SLN plays a pivotal role in whole-body energy metabolism. Using genetically altered mouse models, Maurya et al. [22, 23] have demonstrated that mice with loss of SLN were prone to gain weight, whereas skeletal muscle-specific overexpression of SLN protected mice from developing obesity. The mechanism leading to this response has been reported to involve alteration in cytosolic Ca^2+^ and subsequent enhancement in muscle oxidative metabolism and mitochondrial biogenesis [22–25]. Given the reported effects of SLN on oxidative -and whole-body energy metabolism, manipulation of SERCA via SLN provide a potential therapeutic approach for the treatment of obesity.

However, despite the recent progress in understanding the metabolic role of SLN in skeletal muscles, most studies investigating its role in oxidative metabolism have been carried out in small animals and rodents. Unlike rodents, thermogenesis in large mammals (including humans) are more reliant on skeletal muscle and the expression of SLN is also significantly greater relative to rodents [10]. This leaves a gap in understanding the contribution of SLN to human skeletal muscle metabolism. Therefore, this study was undertaken to further explore the role of SLN for human skeletal muscle energy metabolism. The main objective of this study was to investigate how SLN depletion impacts substrate utilization in human skeletal muscle cells.

## Materials and methods

### Materials

Corning^®^ CellBIND^®^ tissue culture plates were from Corning (Schiphol-Rijk, the Netherlands). Dulbecco’s Modified Eagle’s Medium (DMEM)-Glutamax™ high and low glucose, Dulbecco’s Phosphate Buffered Saline (DPBS; without Ca^2+^ and Mg^2+^), penicillin-streptomycin (10,000 IE/ml), amphotericin B, human epidermal growth factor (hEGF), trypsin-EDTA, and foetal bovine serum (FBS) were purchased from Thermo Fisher Scientific (Waltham, MA, US). Pierce™ BCA Protein Assay Kit, Power SYBR^®^ Green PCR Master Mix, TaqMan reverse transcription kit reagents, High-Capacity cDNA Reverse Transcription Kit, MicroAmp^®^ Optical 96-well Reaction Plate, MicroAmp^®^ Optical Adhesive Film, primers for TaqMan PCR, Nunc™ Cell Culture Treated Flasks with Filter Caps, and GeneJET Plasmid Maxiprep Kit were also purchased from Thermo Fisher Scientific (Waltham, MA, US). Insulin (Actrapid^®^ Penfill^®^100 IE/ml) was from NovoNordisk (Bagsvaerd, Denmark). pMD2.G and psPAX2 were generously provided by Addgene’s non-profit repository (Watertown, MA, US) after deposition by Didier Trono on behalf of his lab. Human GIPZ SLN shRNA viral Particle Starter Kit (VGH5526-EG6588, clone-ID V2LHS_407264 and V2LHS_153115) and GIPZ shRNA empty vector scramble control (RHS4349) were from Horizon Discovery (Cambridge, UK). Polyvinylidene fluoride (PVDF) disk filter was purchased from VWR (Radnor, PA, USA). D-[^14^C(U)]glucose (3.0 mCi/mmol), [1-^14^C]oleic acid (OA, 59.0 mCi/mmol) and [1-^14^C]acetic acid (50.5 mCi/mmol) were from PerkinElmer NEN^®^ (Boston, MA, US). Ultima Gold™ XR, Pico Prias 6 ml PE vials, 96-well Isoplate^®^, UniFilter^®^-96 GF/B microplates, 96-well ScintiPlate^®^-96 Tissue Culture plates and TopSeal^®^-A transparent film were obtained from PerkinElmer (Shelton, CT, US). 4-(2-hydroxyethyl)-1-piperazineethanesulfonic acid (HEPES), β-mercaptoethanol, dimethyl sulfoxide (DMSO), bovine serum albumin (BSA), dexamethasone, gentamicin, L-glutamine, L-carnitine, trypan blue 0.4% solution, D-glucose, oleic acid (OA, 18:1, n-9), carbonyl cyanide 4-(trifluromethoxy)phenylhydrazone (FCCP) and Oil red O were obtained from Sigma-Aldrich (St. Louis, MO, US). QIAshredder and RNeasy Mini Kit were from QIAGEN (Venlo, the Netherlands). Thin-layer chromatography plates were purchased from Merck (Darmstadt, Germany). Free fatty acids (FFA, 2 mg/ml), cholesterol ester (CE, 2 mg/ml) and mono-, di-, triacylglycerol mix (4 mg/ml) were from Supelco (Bellefonte, PA, US). CellTiter 96^®^ AQueous One Solution Cell Proliferation Assay (MTS) Kit was purchased from Promega (Madison, WI, US).

### Methods

#### Production of lentivirus particles

Glycerol stocks were prepared of the pMD2.G envelope plasmid and psPAX2 packaging plasmid. Plasmid DNA was isolated from pMD2.G, psPAX2 and scramble (SCR) shRNAs. Thereafter, a standard protocol [26] for lentivirus particle production through transient transfection of HEK293T cells was used to generate lentiviral particles containing scramble control encoding empty vector shRNA. In brief, HEK293T cells were seeded at a density of 3.5 × 10^6^ cells per 75 cm^2^ NUNC flask and incubated in a humidified atmosphere containing 5% CO_2_ overnight. The cells were then transiently co-transfected with transfer vector plasmid (encoding non-silencing shRNA) in conjunction with a second-generation envelope plasmid (pMD2.G) and a second-generation packaging plasmid (psPAX2). After 8 h, the media was replaced with fresh serum-free DMEM media and incubated for an additional 48 h. To collect the lentivirus particles, the media from 72 to 96 h post transfection were pooled and passed through a 0.45 μm pore size PVDF disk filter remove cell debris and mixed with polyethylene glycol 8000 solution overnight at 4°C. The viral particles were then precipitated at 1600×*g* at 4°C for 1 h and resuspended in PBS before storing at − 80°C until further use.

#### In vitro shRNA transduction of human myoblasts and cell culturing

Primary human satellite cells were isolated from biopsies obtained from *musculus vastus lateralis* of six healthy male donors as previously described [27]. Donors were 23.8 (± 2.4) years old, body mass index 23.9 (± 2.5) kg/m^2^, with fasting glucose levels of 4.7 (± 0.5) mmol/l. Muscle cells obtained from these six donors were then mixed and seeded at a density of 7.5 × 10^5^ cells per 75 cm^2^ NUNC flask using PromoCell medium supplemented with PromoCell Supplement Mix, penicillin (25 IU), streptomycin (25 µg/ml) and amphotericin B (1.25 µg/ml). When a confluence of 30–40% was attained, cells were transduced with SLN shRNA lentivirus particles (V3LHS_407264 and V2LHS_153115) or control (SCR non-silencing shRNA) at a multiplicity of infection (MOI) of 10 in serum- and antibiotic-free medium. The target sequence for shRNA against SLN used in this study were 5’-ACAATATCTGCTATGCTGT-3’ and 5’-CCAGGGAACGGTAGATTTA-3’. 72 h after viral infection, the transduced cells were selected in 0.5 µg/ml puromycin to generate stable cell lines. The cells were then cultured to 80–90% confluence and frozen down in liquid nitrogen until further use.

Before each planned experiment, transduced myoblasts were taken up from liquid nitrogen storage and maintained in DMEM-Glutamax™ (5.5 mmol/l glucose) medium supplemented with 10% FBS, HEPES (25 mmol/l), gentamicin (50 ng/ml), penicillin (25 IU), streptomycin (25 µg/ml), amphotericin B (1.25 µg/ml), hEGF (10 ng/ml), dexamethasone (0.39 µg/ml), and 0.05% BSA. When cells reached ∼80% confluence, differentiation of myoblasts into multinucleated myotubes was initiated by replacing growth medium with DMEM-Glutamax™ (5.5 mmol/l glucose) medium containing 2% FBS and 25 pmol/l insulin. The cells were cultured at 37°C in a humidified 5% CO_2_ atmosphere, and the medium was changed regularly every 2–3 days. Experiments were carried out 7–8 days after the induction of cell differentiation.

#### Substrate oxidation assay

Lentivirus transduced skeletal muscle cells were cultured in 96-well CellBIND^®^ microplates. The cells were then given D-[^14^C(U)]glucose (0.5 µCi/ml, 200 µmol/l) or [1-^14^C]OA (0.5 µCi/ml, 100 µmol/l) substrate during 4 h CO_2_ trapping as described previously [28]. The glucose substrate was prepared in DPBS supplemented with HEPES (10 mmol/l) and BSA (10 µmol/l), whereas the OA substrate was added in DPBS containing HEPES (10 mmol/l), BSA (6.8 µmol/l) and L-carnitine (1 mmol/l). Following tapping, the ^14^CO_2_ produced by the cells and cell-associated (CA) radioactivity was measured using a 2450 MicroBeta^2^ liquid scintillation counter (PerkinElmer). Protein concentration in each well was determined by use of the Pierce BCA Protein Assay Kit to relate the ^14^C-labeled CO_2_ and CA data to cellular protein content.

#### Acid-soluble metabolites (ASMs)

Incomplete fatty acid oxidation (β-oxidation), assessed as ASMs, was measured by acidic precipitation of the radiolabeled incubation medium as described previously [29]. Briefly, 100 µl of the medium containing [1-^14^C]OA from the substrate oxidation assay was transferred to a new tube and precipitated with 30 µl of 6% BSA and 300 µl of 1 M perchloric acid (HClO_4_). Following centrifugation at 10.000 rpm for 10 min, 200 µl of the supernatant was counted by liquid scintillation (Packard Tri-Carb 1900 TR, PerkinElmer).

#### Scintillation proximity assay (SPA)

SPA was performed as previously described [28] to measure the uptake and accumulation of radiolabeled oleic acid substrate by adherent cells. In this study, skeletal muscle cells with and without SLN knockdown (SLN-KD) were cultured on ScintiPlate^®^-96 Tissue Culture plates. Cells were then exposed to [1-^14^C]OA (0.5 µCi/ml, 100 µmol/l) in DMEM medium without phenol red and the accumulation of OA was monitored up to 8 h (at 0, 2, 4, 6, and 8 h) using a 2450 MicroBeta^2^ liquid scintillation counter (PerkinElmer). After the last measurement at 8 h, the cells were washed twice with 0.5% BSA in DPBS with Ca^2+^ and Mg^2+^ and harvested in 0.1% SDS. Protein content in the lysates was measured by Pierce BCA Protein Assay kit using a VICTOR™ *X4* Multilabel Plate Reader (PerkinElmer). Data were related to cellular protein content.

#### Measurements of oxygen consumption

Skeletal muscle cells with and without SLN-KD were plated and cultured on a XF24-well culture plate (Agilent). Before the measurement, culture medium was removed and replaced by assay medium (Agilent) supplemented with 5.5 mmol/l glucose, 1 mmol/l sodium pyruvate and 5 mmol/ HEPES for 1 h at 37 °C. Using the Seahorse XF24 bioanalyzer (Agilent, Wilmington, DE, US), the mitochondrial oxygen consumption rate (OCR), that serve as an indicator of mitochondrial respiration (OXPHOS), was recorded over time following sequential injection of 5 µmol/l oligomycin, 3 µmol/l FCCP and 1 µmol/l rotenone. At the end of the assay, total protein content was measured using Pierce BCA Protein Assay kit and related to OCR values.

#### Lipid distribution

SLN-KD and control (SCR) muscle cells were cultured on 12-well culture plates and incubated with [1-^14^C]OA (0.5 µCi/ml, 100 µmol/l) for 4 h or [1-^14^C]acetic acid (1 µCi/ml, 100 µmol/l) for 24 h. The cells were then washed twice with PBS and harvested in 0.1% SDS. Cellular lipids were extracted with chloroform-methanol (2:1, v/v, Folch extraction) and 0.9% sodium chloride solution (pH 2) as previously described [30, 31]. The radiolabeled extracted lipids were re-dissolved in hexane and separated by thin-layer chromatography. A non-polar solvent mixture of hexane:diethyl ether:acetic acid (65:35:1, v/v/v) and iodine vapor was used to separate the lipids and visualize the lipid bands, respectively. The radioactivity of each bands was quantified by liquid scintillation (Packard Tri-Carb 1900 TR, PerkinElmer) and the amount of lipids was related to cell protein content determined by Pierce BCA Protein Assay kit.

#### MTS cell proliferation assay

Control and SLN-KD cells were seeded at a density of 1 × 10^4^ cells per well in a 96-well CellBIND^®^ microplate. The number of viable cells was determined using a calorimetric CellTiter 96^®^ AQueous One Solution Cell Proliferation Assay according to the manufacturer’s protocol. Briefly, after 7 days of differentiation, MTS reagent containing tetrazolium salt MTS (3-(4,5-dimethylthiazol-2-yl)-5-(3-carboxymethoxyphenyl)-2-(4-sulfophenyl)-2 H-tetrazolium) and phenazine ethosulfate (PES) was added to the cells and incubated further in a humidified atmosphere with 5% CO_2_ at 37 °C for 1 h. The MTS tetrazolium compound is reduced enzymatically by the viable cells into formazan product, whose absorbance at 450 nm is assumed to be directly proportional to the number of viable cells in culture.

#### Oil red O staining

Human myotubes with and without SLN-KD were cultured on 12-well culture plate and preincubated with 200 µM OA for the last 24 h of the differentiation period. The cells were then washed with PBS and fixed with 4% paraformaldehyde for 15 min before rinsed twice with PBS. Afterwards, the myotubes were stained with 0.5% Oil red O (in isopropanol) for 15 min followed by more rinsing steps with PBS and deionized water. Lipid droplets were then visualized by light microscopy (Olympus, Tokyo, Japan) and images were processed using ImageJ software (https://imagej.nih.gov/ij/) and analyzed as described by Deutsch et al. [32].

#### Real time qPCR

Total RNA from cultured transduced cells was extracted using QIAGEN RNeasy Mini Kit according to the manufacturer’s instructions. The RNA was reverse-transcribed into cDNA with High-Capacity cDNA Reverse Transcription Kit and TaqMan Reverse Transcription Reagents using a PerkinElmer Thermal Cycler 9600 (25 °C for 10 min, 37 °C for 80 min and 85 °C for 5 min). The resulting cDNA was subjected to PCR using SYBR^®^ Green Master-mix and primers designed using Primer Express^®^ (Thermo Fisher Scientific). The primers used in this study are listed in Table 1. To quantify mRNA expression levels, qPCR was performed using a StepOnePlus™ Real-Time PCR system (Thermo Fisher Scientific). All assays were run for 44 cycles (95 °C for 15 s followed by 60 °C for 60 s). The transcription levels were normalized to the housekeeping control gene ribosomal protein lateral stalk subunit P0 (*RPLP0*). Normalization to another housekeeping control gene glyceralderhyd-3-phosphate dehydrogenase (*GAPDH*) gave similar results as *RPLP0*.

**Table 1:** Forward and reverse primers used for PCR

Gene	Forward	Reverse
SLN	CCTTGGTGTGCCCTCAGAA	CCGGGTGTTTATCCCCATT
PGC1α	AAAGGATGCGCTCTCGTTCA	TCTACTGCCTGGAGACCTTGATC
FOXO1	GTGTTGCCCAACCAAAGCTT	CTCAGCCTGACACCCAGCTAT
CYC1	CTGCCAACAACGGAGCATT	CGTGAGCAGGGAGAAGACGTA
CPT1B	GAGGCCTCAATGACCAGAATG	GTGGACTCGCTGGTACAGGAA
CD36/FAT	AGTCACTGCGACATGATTAATGGT	CTGCAATACCTGGCTTTTCTCAA
SCD1	CCGCTGGCACATCAAC	ATGGCGGCCTTGGAGACT
FAS	GAACTCCTTGGCGGAAGAGA	GTTCTGAGAAAGGTCGAATTTGC
PDK4	TTTCCAGAACCAACCAATTCACA	TGCCCGCATTGCATTCTTA
RPLP0	CCATTCTATCATCAACGGGTACAA	AGCAAGTGGGAAGGTGTAATCC
GAPDH	TGCACCACCACCTGCTTAGC	GGCATGGACTGTGGTCATGAG

#### Statistical analysis

All values are presented as mean ± SEM in nmol/mg cell protein unless stated otherwise in the figure legends. The value *n* represents the number of individual experiments with 4–16 wells for each condition in each experiment. Statistical analyses and graphical presentations were performed using GraphPad Prism 8.0.1 for Windows (GraphPad Software Inc., San Diego, CA, US). The difference between the groups were compared using unpaired Student’s *t* test. *P* < 0.05 was considered significant.

## Results

### Sarcolipin (*SLN*) expression, cell viability and regulation of target genes involved in metabolism

**Fig. 1:**
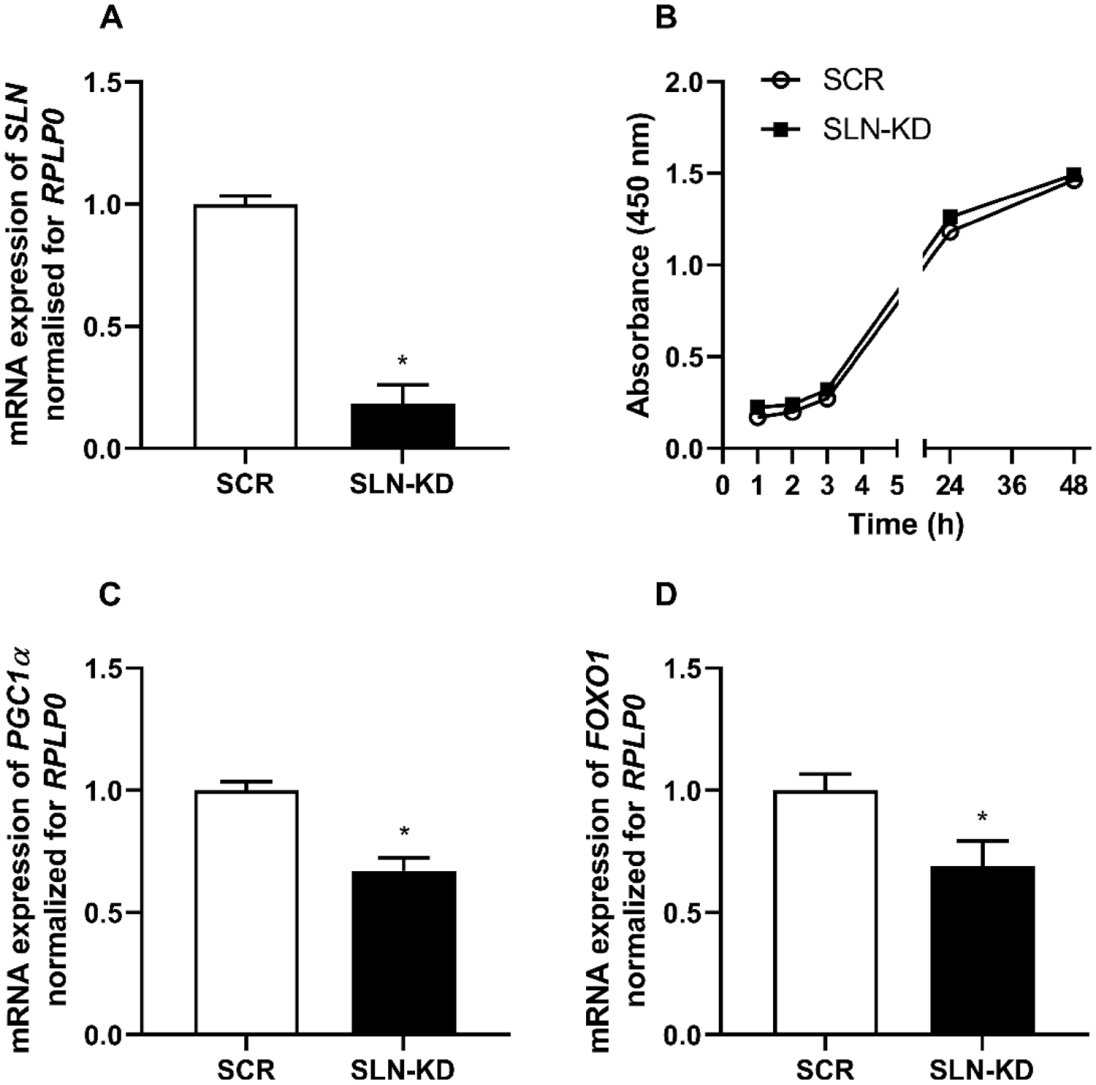
Effects of sarcolipin knockdown (SLN-KD) on cell viability and subsequent regulation of metabolic genes. After 7–8 days of differentiation, control and SLN-KD cells were exposed to MTS reagent or harvested for gene expression analysis. **A** Relative mRNA expression of *SLN*. **B** Cell viability determined by MTS assay. **C** Peroxisome proliferator-activated receptor γ coactivator 1α (*PGC1α*) and **D** forkhead box O1 (*FOXO1*) were measured by qPCR. Results are presented as mean ± SEM (*n* = 4–10). *P < 0.05 vs. control myotubes (SCR)

We first determined the mRNA expression levels of SLN in the myotubes and confirmed that 82% SLN knockdown (SLN-KD) was achieved in our cell model (Fig. 1A). A calorimetric method (MTS) assay was then performed to determine the effect of SLN depletion on cell viability. Our result showed no difference between control and SLN-KD cells (Fig. 1B), indicating that knockdown of SLN did not influence the viability of the cells. To further explore the importance of SLN for skeletal muscle energy homeostasis, we examined whether reduction in SLN expression affected the expression of selected genes known to be involved in oxidative metabolism. Data from qPCR analysis revealed that mRNA expression of the master regulator of mitochondrial biogenesis, peroxisome proliferator-activated receptor γ coactivator 1α (*PGC1α*), was reduced in SLN-KD myotubes compared to control myotubes (SCR) (Fig. 1C). The expression of another transcriptional factor with an important role in skeletal muscle glucose and fatty acid metabolism, forkhead box O1 (*FOXO1*), was also observed to be reduced in SLN-KD myotubes (Fig. 1D).

### Mitochondrial respiration and substrate metabolism

**Fig. 2:**
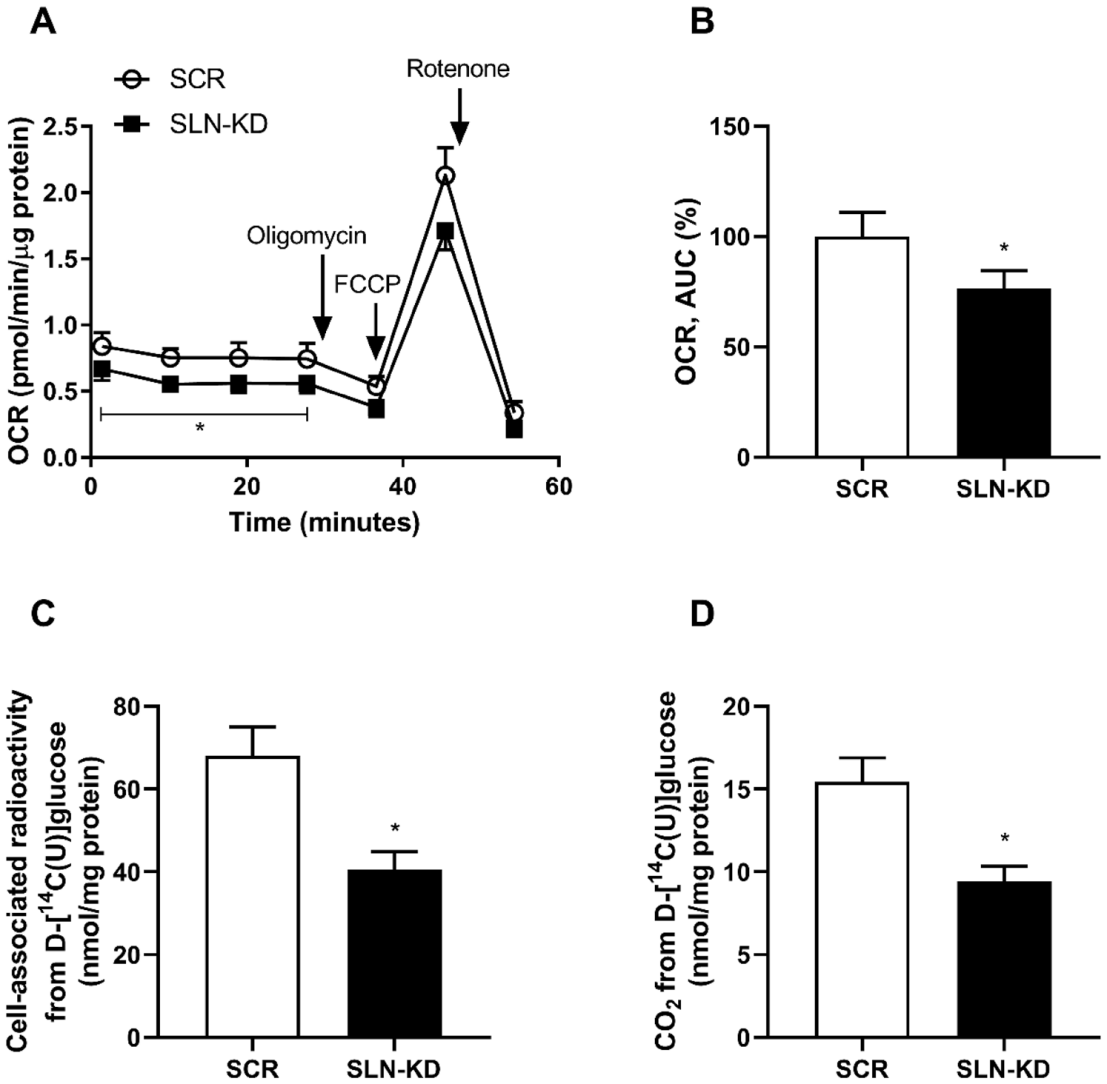
Effects of sarcolipin knockdown (SLN-KD) on mitochondrial function and glucose metabolism. For the assessment of OCR and glucose metabolism, control and SLN-KD cells were cultured and differentiated for 7–8 days. **A** OCR  was measured with a Seahorse XF24 bioanalyzer following a sequential addition of oligomycin, carbonyl cyanide 4-(trifluromethoxy)phenylhydrazone (FCCP) and rotenone. **B** AUC was calculated to compare the total OCR difference between control and SLN-KD cells. Cell-associated radioactivity (**C**) and CO_2_ captured from [^14^C]glucose (**D**) were measured as described in methods. Results are presented as mean ± SEM as nmol/mg cell protein (*n* = 4 in **A** and **B** and *n* = 16 in **C** and **D**). *P < 0.05 vs. control myotubes (SCR)

As it was evident that the expression of crucial genes in energy metabolism (*PGC1α* and *FOXO1*) were significantly downregulated in SLN-KD myotubes, we next examined whether these cells also had altered substrate oxidation and mitochondrial function. After differentiation of SLN-KD and control cells, assessment of oxygen consumption rate (OCR) as a measure of aerobic respiration was performed using a Seahorse bioanalyzer. Real-time analysis of OCR showed that the baseline OCR was reduced in SLN-KD cells (Fig. 2A), indicating lower basal respiration in SLN-KD myotubes. Moreover, SLN-KD myotubes had consistently lower OCR as demonstrated by a 24% reduction in the mean area under the curve (AUC) compared to control myotubes (Fig. 2B). However, other OCR parameters (i.e. basal respiration, proton leak, non-mitochondrial oxygen consumption and ATP production) were not changed (data not shown). Following these results, we further examined the ability of these myotubes to utilize glucose as a substrate by exposing the myotubes to [^14^C]glucose for 4 h. The remaining cell-associated (CA) radioactivity in myotubes (Fig. 2C) and the complete oxidation, measured as the amount of ^14^CO_2_ produced from cellular respiration were observed to be markedly reduced in myotubes with SLN depletion (Fig. 2D).

**Fig. 3:**
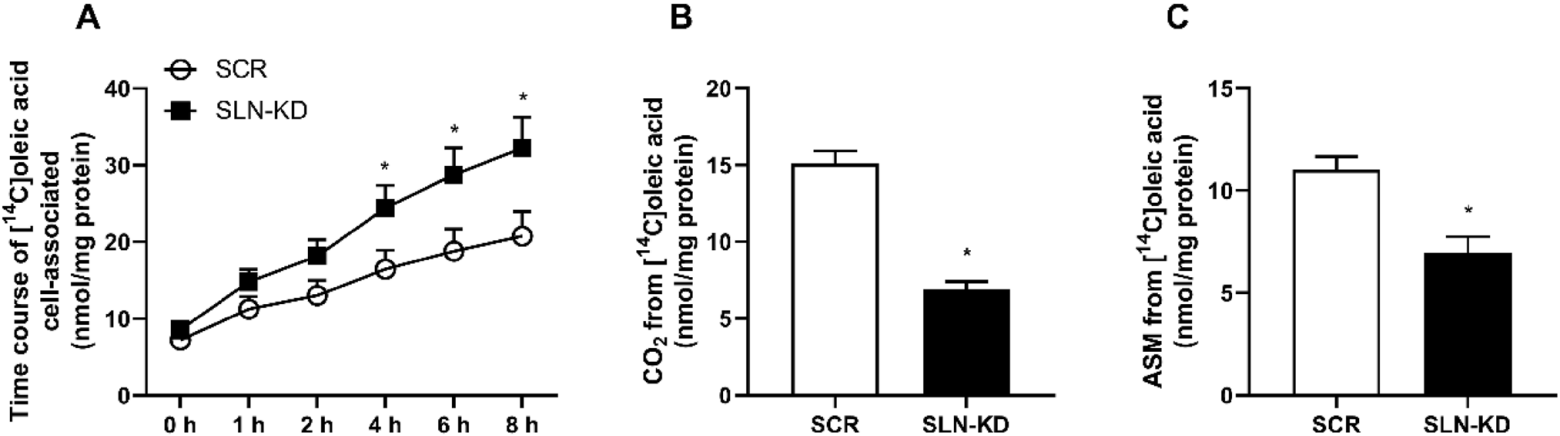
Effects of sarcolipin knockdown (SLN-KD) on accumulation and oxidation of OA. After 7–8 days of differentiation, control and SLN-KD cells were exposed to [^14^C]OA (0.5 µCi/ml, 100 µmol/l) for determination of cellular accumulation and OA oxidation in these myotubes. **A** [^14^C]OA accumulation over 0–8 h. **B** Basal complete oxidation of [^14^C]OA. **C** The incomplete β-oxidation of [^14^C]OA measured as acid soluble metabolites (ASMs). Results are presented as mean ± SEM (*n* = 12 in **A** and *n* = 16 in **B** and **C**). *P < 0.05 vs. control myotubes (SCR)

In addition to measuring glucose utilization, we performed various analysis to investigate how fatty acid metabolism was affected as a consequence of reduced SLN function. Myotubes with or without SLN depletion were differentiated and exposed to [^14^C]OA, before accumulation of OA, complete oxidation (the amount of CO_2_ formed) and incomplete OA oxidation, determined by the amount of acid soluble metabolites (ASM), were measured. As shown in Fig. 3A, myotubes with SLN depletion showed increased accumulation of [^14^C]OA over the time course of 8 h incubation compared to control cells, and the accumulation was significantly higher at all time points from 4 h. Moreover, the ability of SLN-KD myotubes to oxidize OA was markedly diminished (Fig. 3B). Formation of ASM was also observed to be reduced in SLN-KD cells compared to control myotubes, despite a much higher uptake of OA (Fig. 3C).

### Lipid deposition and distribution from oleic acid and de novo lipogenesis

**Fig. 4:**
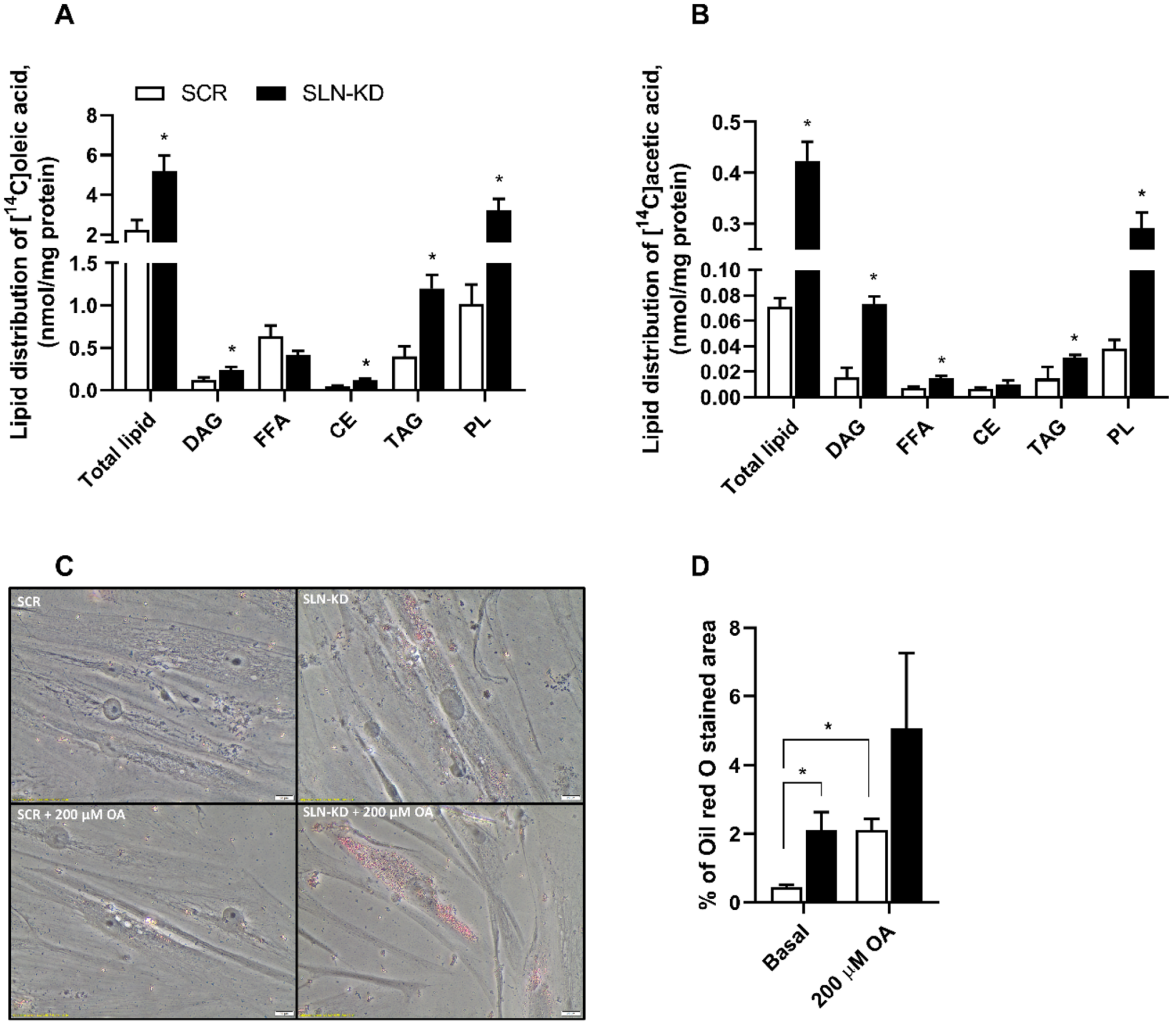
Effects of sarcolipin knockdown (SLN-KD) in primary human myotubes on intramyocellular lipid distribution and de novo lipogenesis. Control and SLN-KD cells were differentiated and incubated with [^14^C]oleic acid (OA, 0.5 µCi/ml, 100 µmol/l) and [^14^C]acetic acid (1 µCi/ml, 100 µmol/l) for 4 and 24 h, respectively. Incorporation of radioactivity into various lipid classes was measured by thin-layer chromatography, as described in methods. Lipid distribution of [^14^C]OA (**A**) and [^14^C]acetic acid (**B**). Additionally, transduced cells were stained with Oil red O to visualize intracellular lipid droplet deposition at basal and following 200 µM OA pretreatment for 24 h. **C** Representative images of Oil red O staining at *X* 20 magnification (scale bar 20 μm) and **D** percentage of positively-stained area calculated using ImageJ. Results are presented as mean ± SEM as nmol/mg cell protein (*n* = 9 in **A** and *n* = 8 in **B**) and as % stained area (*n* = 6–7 in **C** and **D**). *P < 0.05 vs. control myotubes (SCR). *FFA* free fatty acids, *DAG* diacylglycerol, *TAG* triacylglycerol, *CE* cholesteryl ester, *PL* phospholipids

Our results suggest that the ability to utilize energy substrates was diminished in SLN-KD cells. Therefore, we performed additional experiments to further evaluate whether reduced SLN function had also an impact on intracellular lipid distribution. Indeed, incorporation of [^14^C]OA into various lipid classes after 4 h incubation showed that total cellular lipid, cholesteryl ester (CE), triacylglycerol (TAG), diacylglycerol (DAG), and phospholipids (PL) were increased in SLN-KD myotubes compared with control cells, whereas the level of unesterified OA (FFA) was not affected (Fig. 4A). Furthermore, we investigated the incorporation of [^14^C]acetic acid into different lipids in order to study the role of SLN in *de novo* lipogenesis. Following 24 h incubation of SLN-KD and control myotubes, formation of FFA and complex lipids from [^14^C]acetic acid was measured. Compared to control cells, myotubes with SLN-KD resulted in higher levels of cellular total lipid, as well as DAG, TAG, FFA, and PL from acetic acid, whereas the levels of CE remained unchanged (Fig. 4B). In addition, Oil red O staining of the cells demonstrated that SLN-depleted myotubes had significantly higher accumulation of lipid droplets under basal conditions (Fig. 4C, D). SLN-KD cells also seems accumulate more lipid droplets when exposed to an overnight fatty acid load (Fig. 4C, D**)**.

### Alterations in gene expression

**Fig. 5:**
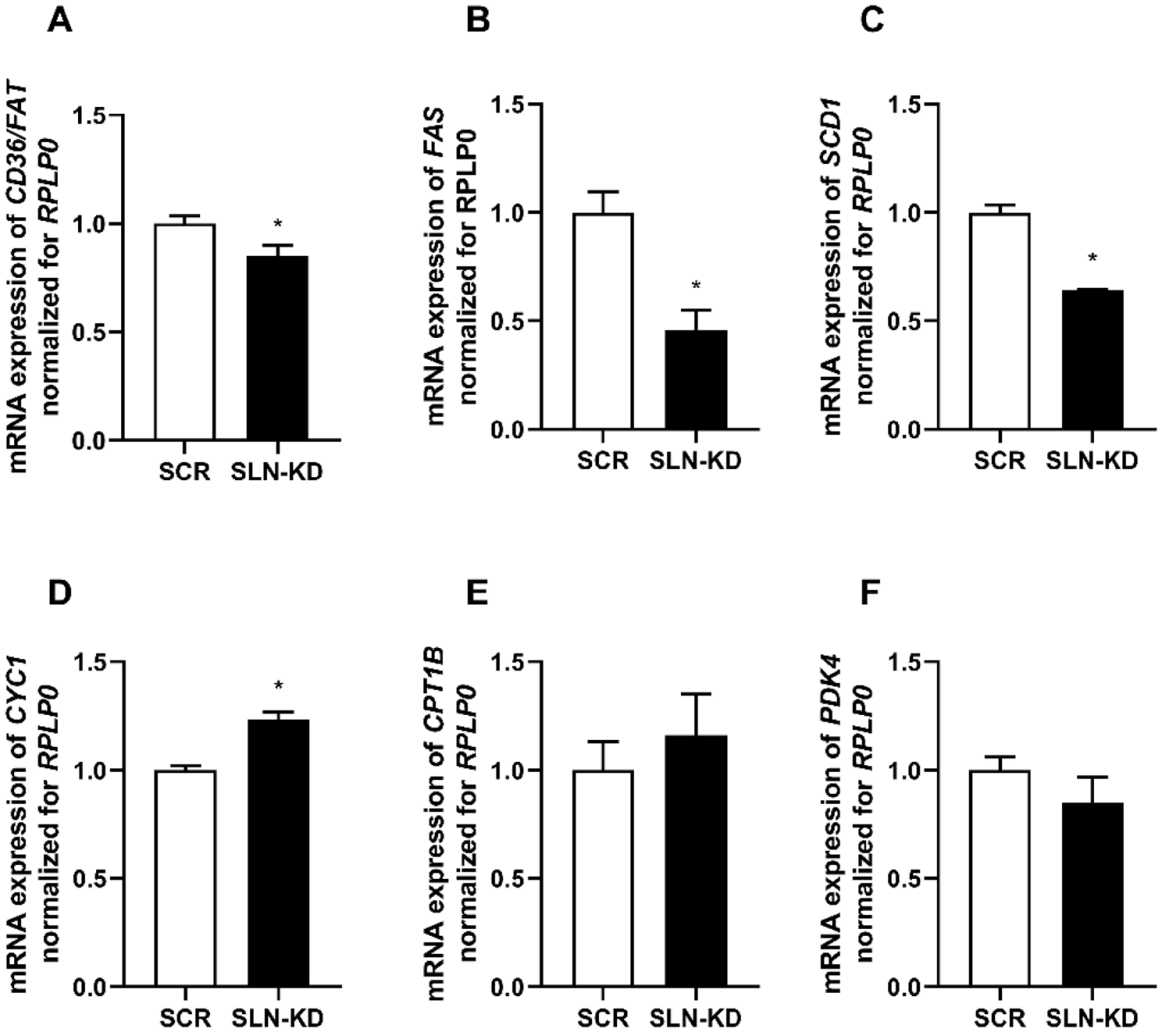
Changes in mRNA expression due to sarcolipin knockdown. RNA was isolated from control and sarcolipin knockdown (SLN-KD) myotubes following 7–8 days of differentiation. Expressions of selected genes were assessed by qPCR. Values from qPCR were corrected for the housekeeping gene ribosomal protein lateral stalk subunit P0 (*RPLP0*). Relative mRNA expression levels of genes related to lipid storage (**A**–**C**) and mitochondria-related genes (**D**–**F**) in SLN-KD and control cells. Results are presented as mean ± SEM (*n* = 4–9). *P < 0.05 vs. control myotubes (SCR). *SCD1* stearoyl-CoA desaturase 1, *CPT1B* carnitine palmitoyltransferase 1B, *PDK4* pyruvate dehydrogenase kinase 4, *CYC1* cytochrome c1, *CD36/FAT* fatty acid transporter, *FAS* fatty acid synthase

Finally, to investigate possible causes to the metabolic perturbations in cells with SLN depletion, the expression of certain genes involved in substrate metabolism were measured. As shown in Fig. 5, in cells with knockdown of SLN we found reduced expression of the fatty acid transporter *CD36/FAT* (Fig. 5A). Surprisingly, the mRNA levels of fatty acid synthase (*FAS*) (Fig. 5B) and stearoyl-CoA desaturase 1 (*SCD1*) (Fig. 5C), which are considered key rate-controlling enzymes in lipid synthesis were also reduced. In contrast, the expression of an enzyme involved in electron transfer during oxidative phosphorylation, cytochrome c1 (*CYC1*), was observed to be increased in SLN-KD myotubes compared with control cells (Fig. 5D). The expression levels of carnitine palmitoyltransferase 1B (*CPT1B*) and pyruvate dehydrogenase kinase 4 (*PDK4*) were not significantly different between the two types of myotubes (Fig. 5E, F).

## Discussion

The present study provides evidence that SLN plays an important role in regulating skeletal muscle energy metabolism. Although many thermogenic and metabolic properties of SLN have been reported, there is limited knowledge on the function of SLN for human skeletal muscle oxidative metabolism. Thus, to the best of our knowledge, this study is the first to characterize the metabolic effects of reduced SLN expression in differentiated human myotubes. Following generation of human muscle cells with stable SLN-KD, cell viability and some fundamental metabolic parameters were assessed. The findings revealed that depletion of SLN did not influence cell viability. However, we observed a significant reduction in complete glucose and OA oxidation to carbon dioxide. The reduction in complete OA oxidation was also accompanied by diminished fatty acid β-oxidation. Moreover, lipid accumulation, lipid distribution as well as *de novo* fatty acid synthesis were increased in SLN-KD myotubes compared to control cells.

Skeletal muscle plays a key role in temperature homeostasis and is involved in heat generation through shivering and NST [9, 11]. Increasing energy consumption in skeletal muscles through SERCA-mediated thermogenesis can affect whole-body energy metabolism and might be an effective strategy to counteract weight gain [11]. Previous studies have shown that SLN plays a prominent thermoregulatory role by inducing futile cycling of the SERCA pump leading to slippage of Ca^2+^ back to the cytosol promoting an increase in ATP hydrolysis and heat production [17, 19, 20, 22, 33]. As a mediator of NST in muscle, SLN has become a topic of interest with studies showing that its genetic ablation in a mouse model has been linked with diet-induced obesity, glucose intolerance, as well as hypothermia when challenged with acute cold [17, 34]. On the other hand, skeletal muscle-specific SLN overexpression in mice increased basal metabolic rate and induced resistance against diet-induced obesity [22, 23]. These findings have highlighted the importance of uncoupling SERCA by SLN in regulating energy expenditure and contributing to whole-body energy metabolism.

In the present study, we addressed the role of SLN in muscle energy metabolism by generating a SLN-KD human skeletal muscle cell model. In vitro cell culturing is a suitable model system to conduct genetic knockdown experiments that involves measurements of cellular function. In addition to the controlled extracellular environment, the genetic background of these skeletal muscle cells is highly relevant to mimic closely intact human skeletal muscle [35] and thus can be an important model system when aiming to translate data from in vitro to in vivo.

One of our primary objectives was to assess whether the ability of the cells to oxidize energy substrates was affected due to SLN depletion. Here, we discovered that the complete oxidation of both glucose and OA in SLN-KD human myotubes were markedly diminished with a concomitant decrease in mitochondrial fatty acid β-oxidation. These results were supported by the consistently lower basal oxygen consumption observed by respirometry, indicating that the mitochondrial efficacy in SLN-KD cells were reduced, possibly attributed to decreased mitochondrial proton uncoupling or ATP demand. Surprisingly, mRNA expression of heme-containing component of complex III (*CYC1*) in the respiratory chain, was found to be elevated in SLN-KD cells. We cannot explain the increased expression of *CYC1*, while basal respiration as well as mitochondrial fatty acid β-oxidation were reduced. However, the contribution of other mitochondrial complex chains must not be excluded for the reduction in mitochondrial function. Indeed, our findings are generally in line with previously reported data from whole-body SLN knockout mice [23], where ablation of SLN resulted in a reduction of fatty acid oxidation and decreased oxygen consumption. Another study by Paran et al. [36] also showed that lentiviral knockdown of SLN reduced basal respiration in both lean and obese human skeletal muscle cells. Thus, the results of the present study offer additional support for the critical role of SLN in regulating mitochondrial oxidative capacity and basal energy expenditure.

Moreover, using primary muscle cells derived from SLN overexpressing and wild-type (WT) control mice, Maurya et al. [22, 23] demonstrated that SLN activated Ca^2+^/calmodulin-dependent protein kinase II (CamKII) and subsequent recruitment of PGC1α to promote mitochondrial biogenesis and enhance oxidative metabolism. These studies have also shown that the attenuated oxidative capacity in the absence of SLN expression was mainly due to reduced expression of fatty acid transporters and mitochondrial oxidative enzymes [22, 23]. On the other hand, in SLN knockout mice, re-expression of SLN fully restored muscle-based thermogenesis [17], and in myotubes from SLN knockout mice, the phenotype was rescued by restoring respiratory capacity as well as PGC1α expression to WT control levels [23]. This prompted us to examine the mRNA expression of selected genes involved in skeletal muscle mitochondrial oxidative capacity and substrate metabolism. Indeed, we observed a significant downregulation of the transcriptional coactivator *PGC1α* in myotubes with SLN knockdown. In skeletal muscle, PGC1α can induce various responses including adaptive thermogenesis, oxidative phosphorylation, and mitochondrial biogenesis [37]. Hence, metabolic perturbations observed due to knockdown of SLN may therefore be attributed to the reduced expression of this key regulator of energy metabolism.

Being a versatile coactivator, PGC1α can interact with various transcriptional factors, including FOXO1, in order to promote distinct biological responses [37–39]. It has been shown that energy deprived states such as fasting, calorie restriction, and exercise upregulates the expression of FOXO1 in skeletal muscle [40]. This suggests that FOXO1 may promote skeletal muscle adaptations to changes in energy metabolism in a similar way as PGC1α. Evidently, FOXO1 can regulate the carbohydrate/lipid metabolic shift in skeletal muscle during periods of starvation by inducing upregulation of PDK4, lipoprotein lipase and the fatty acid translocase CD36/FAT (reviewed in [38]). In SLN-KD myotubes we found reduced mRNA expression of *FOXO1*. Though we found no difference in *PDK4* expression between SLN-KD and control myotubes, the reduced expression of *CD36/FAT* observed in SLN-KD cells could be a result of *FOXO1* downregulation in these cells. Therefore, the uptake and intracellular transport of OA appeared not to be strictly mediated by CD36/FAT, indicating that the increased fatty acid accumulation observed in SLN depleted cells might involve other fatty acid transport mechanisms. These data suggests that reduced *FOXO1* activity, at least in part, could be accounted for perturbation of fatty acid metabolism found in SLN-KD myotubes.

Further, it has been shown that the oxidation of glucose and fatty acids was impaired at the level of the mitochondria in skeletal muscle of individuals with obesity and type 2 diabetes [41, 42]. Abnormal fatty acid metabolism due to depressed oxidative metabolism in muscle leads to excess accumulation of TAG and other lipid metabolites such as CE and DAG within the skeletal muscle and attenuate insulin signaling [43, 44]. Evidence from SLN knockout mice has demonstrated a significant accumulation of lipid droplets in muscle compared to WT animals, indicating depressed lipid utilization [23]. Conversely, muscle from SLN overexpressing mice showed lower intramuscular concentration of CE, DAG and acylcarnitines compared to WT [23]. Indeed, we found that cellular accumulation of OA as well as intracellular incorporation of labeled OA into CE and DAG was significantly increased in myotubes with SLN knockdown. Additionally, the amount of TAG and PL from OA was also elevated due to reduced SLN function. Detection of neutral lipid deposits using Oil red O staining also showed increased lipid accumulation in SLN-KD cells under basal condition, which further strengthens the evidence that SLN malfunction could be linked to metabolic impairments associated with obesity and type 2 diabetes.

In addition to exogenous uptake by skeletal muscle, fatty acids are also derived from excess carbohydrates through the process of *de novo* fatty acid biosynthesis (lipogenesis) [45, 46]. Although this is not considered to be a major contributor to total fatty acid flux in skeletal muscle [45], *de novo* lipogenesis may lead to adverse metabolic consequences when dysregulated. Using labeled acetic acid as a substrate, we discovered that formation of complex lipids (i.e. DAG, TAG, and PL) and FFA were enhanced in myotubes with SLN depletion. The increased accumulation of lipids in the absence of exogenously supplied fatty acids together with reduced glucose and fatty acid oxidation found in SLN-KD myotubes are likely to have negative effects associated with obesity, including insulin resistance and dyslipidemia.

To provide a possible explanation for the increased intramyocellular lipid deposition, we measured the expressions of key lipogenic enzymes, FAS and SCD1 [47, 48], which are involved in formation of lipid metabolites via lipogenesis. Surprisingly, knockdown of SLN mediated reduced mRNA expression of both *FAS* and *SCD1*. We can only speculate that this presumably could be a result of diminished fatty acid oxidation by muscle mitochondria or perhaps a compensatory adaptation to the increased risk of lipotoxicity. Interestingly, a recent study by Funai et al. have showed the presence of FAS in the sarcoplasmic reticulum (SR) fraction of mice skeletal muscle [49]. Findings from this particular study also indicated that FAS deficiency can reduce SERCA activity by altering phospholipid composition of the SR, leading to an increase in cytosolic Ca^2+^ and CaMKKβ-dependent activation of AMP-activated protein kinase (AMPK). It is known that activation of AMPK induces metabolic changes including stimulation of glucose and fatty acid catabolism, as well as mitochondrial biogenesis [50]. Reduction of FAS activity may therefore be a way for SLN-KD myotubes to cope with the lipid overload. Moreover, there is evidence suggesting that lack of SCD1 can also be favorable with respect to increasing energy expenditure through thermogenesis and subsequent increase in fatty acid oxidation [47, 51]. Thus, the observed reduction of both *FAS* and *SCD1* expressions could be a compromise in order to compensate for an ineffective SERCA activity in SLN-KD myotubes.

Although our results showed that knockdown of SLN induces metabolic perturbations in human skeletal muscle cells, possibly as a result of ineffective SERCA activity, we were not able to assess the effect of SLN depletion on SERCA Ca^2+^ transport activity in our myotubes. This is a potential limitation of the current study. However, previous studies on cardiac and skeletal muscle homogenates from mice have shown that ablation of SLN increased the apparent affinity of SERCA for Ca^2+^ and the maximum velocity of Ca^2+^ uptake rate, which can further enhance relaxation rates [52, 53].

In summary, we corroborated that SLN plays an important role in skeletal muscle energy metabolism. Cultured myotubes with knockdown of SLN showed markedly diminished glucose and OA utilization, possible due to reduced mitochondrial function. We also showed that both exogenous and endogenous lipid synthesis was increased in SLN-KD myotubes compared to control cells. Moreover, mRNA expression levels of *PGC1α* and *FOXO1* were reduced by SLN depletion, elucidating, at least in part, the molecular basis behind metabolic perturbations observed in SLN-KD myotubes. Interestingly, the activity of key lipogenic enzymes were downregulated, which suggest the possible existence of compensatory mechanisms due to inefficiency of SLN-mediated energy utilization. Collectively, our findings highlight that SLN-based manipulation of energy expenditure in skeletal muscle might be a target to counter obesity and associated metabolic disorders.

## Data Availability

All data generated or analyzed during this study are included in this published article.

## Abbreviations

SERCA: Sarco-/endoplasmic reticulum Ca^2+^-ATPase
OA: Oleic acid
SLN: Sarcolipin
ASM: Acid soluble metabolites
